# Sperm proteins and cancer‐testis antigens are released by the seminiferous tubules in mice and men

**DOI:** 10.1096/fj.202002484R

**Published:** 2021-02-10

**Authors:** Liza O'Donnell, Diane Rebourcet, Laura F. Dagley, Raouda Sgaier, Giuseppe Infusini, Peter J. O'Shaughnessy, Frederic Chalmel, Daniela Fietz, Wolfgang Weidner, Julien M. D. Legrand, Robin M. Hobbs, Robert I. McLachlan, Andrew I. Webb, Adrian Pilatz, Thorsten Diemer, Lee B. Smith, Peter G. Stanton

**Affiliations:** ^1^ Hudson Institute of Medical Research Clayton VIC Australia; ^2^ Department of Molecular and Translational Sciences Monash University Clayton VIC Australia; ^3^ Faculty of Science The University of Newcastle Callaghan NSW Australia; ^4^ Walter and Eliza Hall Institute Parkville VIC Australia; ^5^ Department of Medical Biology University of Melbourne Parkville VIC Australia; ^6^ Department of Urology, Pediatric Urology and Andrology Medical Faculty Justus‐Liebig‐University Giessen UKGM GmbH Giessen Germany; ^7^ Univ Rennes Inserm EHESP Irset (Institut de recherche en santé, environnement et travail) ‐ UMR_S 1085 Rennes France; ^8^ Inserm, EHESP, Irset (Institut de recherche en santé, environnement et travail), UMR_S 1085 University Rennes Rennes France; ^9^ Institute for Veterinary Anatomy, Histology and Embryology Justus‐Liebig‐University Giessen Giessen Germany; ^10^ MRC Centre for Reproductive Health The Queen's Medical Research Institute University of Edinburgh Edinburgh UK

**Keywords:** biomarker, cancer‐testis antigen, interstitial fluid, sperm, testis

## Abstract

Sperm develop from puberty in the seminiferous tubules, inside the blood‐testis barrier to prevent their recognition as “non‐self” by the immune system, and it is widely assumed that human sperm‐specific proteins cannot access the circulatory or immune systems. Sperm‐specific proteins aberrantly expressed in cancer, known as cancer‐testis antigens (CTAs), are often pursued as cancer biomarkers and therapeutic targets based on the assumption they are neoantigens absent from the circulation in healthy men. Here, we identify a wide range of germ cell‐derived and sperm‐specific proteins, including multiple CTAs, that are selectively deposited by the Sertoli cells of the adult mouse and human seminiferous tubules into testicular interstitial fluid (TIF) that is “outside” the blood‐testis barrier. From TIF, the proteins can access the circulatory‐ and immune systems. Disruption of spermatogenesis decreases the abundance of these proteins in mouse TIF, and a sperm‐specific CTA is significantly decreased in TIF from infertile men, suggesting that exposure of certain CTAs to the immune system could depend on fertility status. The results provide a rationale for the development of blood‐based tests useful in the management of male infertility and indicate CTA candidates for cancer immunotherapy and biomarker development that could show sex‐specific and male‐fertility‐related responses.

**Abbreviations**
ACN: acetonitrile
CID: collision‐induced dissociation
CTA: cancer‐testis antigen
DTR: diphtheria toxin receptor
DTX: diphtheria toxin
FA: formic acid
FDR: False discovery rate
iBAQ: intensity‐based absolute quantification
LDHC: lactate dehydrogenase C
MS: mass spectrometry
M‐TESE: microsurgical‐assisted testicular sperm extraction
OA: obstructive azoospermia
PBS: phosphate‐buffered saline
PCA: principle component analysis
PSC: pachytene spermatocytes
PSMs: peptide‐spectrum matches
PTMCs: peritubular myoid cells
rST: round spermatids
SCO: sertoli cell‐only
TIF: testicular interstitial fluid
ZAN: zonadhesin

## 1 INTRODUCTION

Sperms form inside the seminiferous tubules of the testes at puberty, well after the establishment of the immune system in early neonatal life, and developing sperm must be protected from the immune system to prevent their recognition as foreign. Outside the seminiferous tubules, the testicular interstitium contains abundant immune cells, including macrophages, mast cells, and dendritic cells, yet it is a unique immunosuppressed microenvironment by virtue of local immunoregulatory mechanisms.^1^, ^2^, ^3^ Developing sperm are physically sequestered from the interstitium and resident immune cells by the blood‐testis barrier.^1^, ^2^, ^3^, ^4^ Meiotic spermatocytes and post‐meiotic spermatids develop inside the blood‐testis barrier, in a specialized milieu known as the adluminal compartment.^4^, ^5^ Tight junctions between the somatic Sertoli cells of the seminiferous tubules restrict the free passage of proteins into the adluminal compartment and entry of immune cells and antibodies into the tubules.^4^ Immune privilege in the testis is considered to be a combination of the physical sequestration of developing sperm inside the blood‐testis barrier, and local immunomodulatory factors that promote an immune‐suppressed environment.^1^, ^3^, ^5^

A widely held assumption is that proteins specifically expressed by sperm remain inside the blood‐testis barrier,^5^, ^6^ protected from immune system recognition, and prevented from entering the circulation. This assumption has led to an interest in cancer‐testis antigens (CTAs) as potential therapeutic targets and biomarkers of, various cancers.^7^, ^8^, ^9^, ^10^ CTAs are proteins normally only expressed in male germ cells but aberrantly expressed in cancer.^8^, ^9^ Since sperm‐specific CTAs are widely assumed to be restricted within the blood‐testis barrier in healthy men, they are assumed to be neoantigens that will provoke a large immune response and thus considered excellent targets for cancer immunotherapy.^7^, ^11^, ^12^, ^13^, ^14^ Because they are also assumed to be absent from the circulation in healthy men but can be aberrantly expressed in cancer, CTAs are being explored for their utility as circulating cancer biomarkers.^15^, ^16^

Although these assumptions have become accepted wisdom in the wider literature, reproductive biologists have long speculated that not all sperm‐specific proteins remain inside the seminiferous tubules.^6^, ^17^, ^18^, ^19^, ^20^ Vasectomy causes the leakage of sperm from the inflamed epididymis and thus should result in a massive immune response against a wide range of sperm antigens as sperm are recognized by the immune system for the first time. Yet, vasectomy is followed by the generation of an unexpectedly narrow repertoire of sperm autoantibodies, pointing to the existence of immune tolerance to at least some sperm antigens.^20^, ^21^, ^22^ Proof of this concept was achieved in mice, where the sperm‐specific protein and CTA, lactate dehydrogenase 3 (LDH3, also known as LDHC), was shown to promote T regulatory cell (Treg)‐mediated peripheral tolerance but another sperm protein, zonadhesin, was non‐tolerogenic.^23^ These observations suggest that certain sperm proteins are not sequestered inside the seminiferous tubules by the blood‐testis barrier and could encounter the immune system.

However, there has been no in‐depth analysis of germ cell proteins in the fluid outside of the seminiferous tubules in humans or mice. Given the interest in CTAs for immunotherapy and cancer biomarker development, it is imperative to identify whether, and which, germ cell proteins can be deposited by the seminiferous tubules into the surrounding interstitial fluid, particularly in humans. A comprehensive survey of the proteins released by human seminiferous tubules may also provide new opportunities for non‐invasive monitoring of spermatogenic function. To address these issues, we completed the first in‐depth characterization of the mouse and human interstitial fluid proteomes. The interstitial space between the tubules contains testicular interstitial fluid (TIF), comprised of secretions and products from the seminiferous tubules, the interstitial cells, and the circulation.^24^ We identify a wide range of germ cell proteins, including proteins expressed only in sperm and CTAs that are deposited by the seminiferous tubules into the TIF in mice and humans.

## 2 METHODS

### 2.1 Study approval

Mice were housed and bred under standard conditions of care. Experiments were conducted with licensed permission under the UK Animal Scientific Procedures Act (1986), Home Office license number PPL 60/4200.

All human procedures performed were in accordance with the ethical standards of the Institutional and/or National Research Committee and with the 1964 Helsinki Declaration and its later amendments or comparable ethical standards. All patients were counseled preoperatively and gave written informed consent to perform testicular surgery. This study was approved by the local institutional review board (Ethik‐Kommission am FB 11 “Humanmedizin,” Justus‐Liebig‐Universität Giessen; Ref. No. 26/11).

### 2.2 Model of seminiferous tubule cell ablation

The seminiferous epithelium was disrupted in adult mice using a model of acute (1 week) Sertoli cell ablation.^25^, ^26^ This model utilizes transgenic mice expressing diphtheria toxin receptor (DTR) specifically in Sertoli cells driven by *Amh‐cre*.^26^ After one week of diphtheria toxin (DTX) administration, very few Sertoli cells are present in the seminiferous tubules (Supplemental Figure S1). Although spermatogonia, spermatocytes, and elongated spermatids are visible, many are clearly undergoing apoptosis and the mRNA expression of germ cell markers (*Pouf5a1* for spermatogonia, *Spo11* for spermatocytes, and *Tp1,* also known as *Tnp1,* for spermatids) are markedly reduced (Supplemental Figure S1).^25^ At this time, peritubular myoid cells (PTMCs) remain around the tubules but show reduced expression of the PTMC functional marker calponin.^25^ Leydig cell number is unaffected after one week; however, some of these cells eventually undergo apoptosis.^25^ There were minor but significant changes in the expression of inflammatory markers during one week of DTX treatment, but macrophage infiltration was relatively minor (Supplemental Figure S2).^25^, ^26^

### 2.3 Isolation of mouse TIF

Adult male animals (>70 days) Amh‐Cre+/+;iDTR+/+ were used for this study.^25^ Mice were injected with a single dose of 100ng DTX (DTX group, n = 11) or vehicle (control group, n = 12)^25^ and were culled 1 week later using CO_2_ asphyxiation and cervical dislocation. Testes were collected, weighed and TIF was collected as described^27^: briefly, samples were cleaned in cold PBS containing protease inhibitors (cOmplete, Mini, EDTA‐free Protease Inhibitor Cocktail, Roche, UK) and dried on filter tissue. A small incision through the tunica albuginea was made prior to centrifuging the tissues (1000 *g*, 1 minute, 4°C). Each testis was then suspended using sutures, decapsulated, and dipped quickly in three sequential 1.5 mL Eppendorf tubes containing PBS and protease inhibitors. TIF was collected by centrifugation (10 000 *g*, 15 minutes, 4°C) of the pooled sequential collection tubes and the supernatant was stored at −80°C.

### 2.4 Isolation of human TIF

For proteomic analyses, TIF was taken from three men diagnosed as azoospermic due to distal reproductive tract obstruction (obstructive azoospermia, OA); however, all data suggested that their testicular function was normal (Supplemental Dataset 9). Normal histology of their testis was determined by morphological evaluation. Specimens from each testicular incision site (Supplemental Dataset 9) were immediately fixed in Bouin's solution and processed according to routine protocols. The semi‐quantitative score count evaluation of spermatogenesis was performed according to Bergmann and Kliesch.^28^ For each individual retrieval site, the number of tubules containing elongated spermatids is divided by the total number of tubules examined × 10; hence, score values range from 0 to 10. This histologic diagnosis procedure allows patients to be classified into four groups: normal spermatogenesis (score 8‐10), hypospermatogenesis,^1^, ^2^, ^3^, ^4^, ^5^, ^6^, ^7^ predominant tubular atrophy (0.1‐0.9), and Sertoli cell‐only tubules (SCO) (0).^28^

TIF was collected by experienced microsurgeons (TD, WW, AP) from patients undergoing M‐TESE (microsurgical‐assisted testicular sperm extraction) for sperm retrieval, as described.^29^ This procedure uses a midline incision and a microscope with ×15 magnification to incise the tunica albuginea and connective tissue to access the seminiferous tubules. Prior to the dissection of tubules, TIF was recovered adjacent to the tubules by applying gentle pressure on the tissue and collected using a micro‐syringe (1 mL) fitted with a plastic tip. An average of 200‐500 µL TIF collected per testis was immediately snap‐frozen in Eppendorf tubes over dry ice in the operating theater and subsequently stored at −80°.

For Western blotting, TIF was taken from n = 8 men with OA defined as above and from eight men with presumed Sertoli cell‐only phenotype.^28^ Clinical data from the OA and SCO groups, including hormones and testis volumes, are shown in Supplemental Dataset 10A. SCO men had significantly higher levels of FSH than OA men. Spermatogenesis score^28^ for the biopsies taken from each patient is shown in Supplemental Dataset 10B; all SCO patients had biopsy scores of zero and were unable to have sperm retrieved from their testes during surgery.

### 2.5 Proteomics of mouse and human TIF

#### 2.5.1 Trypsin digestion

Protein concentrations were determined by the BCA method (Pierce, Rockford). For mouse TIF, equal amounts of mouse TIF lysate (60 μg) from DTX (n = 11) and control (vehicle‐treated) mice (n = 12) were prepared for mass spectrometry analysis using the FASP protein digestion method^30^ with the following modifications. Protein material was reduced with Tris‐(2‐carboxyethyl)‐phosphine (TCEP, 10 mM final concentration). Eluates were digested with sequence‐grade modified trypsin Gold (Promega, V5280) (1 μg) in 50 mM ammonium bicarbonate (NH4HCO3) and incubated overnight at 37°C. Peptides were eluted with 50 mM NH4HCO3 in two 40 μL sequential washes and acidified in 1% formic acid (final concentration). For the human TIF, 200 μg of protein from three individual OA patients was prepared for mass spectrometry analysis using the USP3 protocol.^31^ We used a 1:1 combination mix of the two types of commercially available carboxylate beads (Sera‐Mag Speed beads, #65152105050250, #45152105050250, Thermo Fisher Scientific). Beads were prepared freshly each time by rinsing with water three times prior to use and stored at 4°C at a stock concentration of 20 μg/μL. Samples were transferred to a 2 mL LoBind deepwell plate (Eppendorf, Hamburg, Germany) and reduced with 2 M dithiothreitol (DTT, 50 mM final conc.) for 1 hour at 37°C. Samples were then alkylated with 1M iodoacetamide (100 mM final conc.) for 30 mins in the dark at room temperature (RT). Samples were quenched with 2M DTT (250 mM final conc.) and 4 μL of the concentrated bead stock carboxylate beads (20 μg/μL) was added to each sample followed by the addition of acetonitrile (ACN) to a final concentration of 70% (v/v). Mixtures were left to incubate upright at RT for 20 mins to allow proteins to precipitate onto the beads. The beads were placed on a magnetic rack and washed twice with 70% ethanol and once with ACN (500 μL washes). ACN was completely evaporated from the plate using a CentriVap (Labconco, Kansas City, MO, USA) prior to the addition of 40 μL of digestion buffer (10% 2‐2‐2‐trifluoroethanol /100 mM NH4HCO3) containing 4 μg Trypsin‐gold (Promega, V5280) and 4 μg Lys‐C (Wako). The plate was briefly sonicated in a water bath to disperse the beads, and the plate was transferred to a ThermoMixer C instrument (Eppendorf) for enzymatic digestion at 37°C for 1 hour (1200 rpm). The supernatant comprising of peptides was then collected from the beads using a magnetic rack (Ambion, Thermo Fisher Scientific) and an additional elution (50 μL of 2% dimethyl sulfoxide, Sigma) was performed on the beads. The eluates were pooled together then equally split across pre‐equilibrated C18 stage tips for sample clean‐up. Briefly, six plugs of C18 resin (3M Empore, 66883‐U) were prepared in 200 μL unfiltered tips, pre‐wetted with 100 μL of methanol followed by sequential washes with 100 μL of 80% acetonitrile (ACN)/5% formic acid (FA), 50% ACN/5% FA and 5% FA. The pooled peptides were then added to the spin tip and the eluate collected into a fresh lo‐bind Eppendorf tube. Bound peptides were washed twice with 5% FA. Elutions (50 μL) were performed sequentially with 50% ACN/5% FA followed by 80% ACN/5% FA and collected into fresh Eppendorf tubes. All spins were performed on a benchtop centrifuge at 500 *g* (1000‐2000 rpm) speeds. The eluates were lyophilized to dryness in MS vials (CentriVap) prior to reconstituting in 40 μL of 5 mM ammonium formate buffer, pH 10 ready for offline peptide fractionation on an HPLC.

#### 2.5.2 Offline HPLC fractionation

Tryptic peptides from each of the three human TIF samples were subjected to high pH reverse‐phase analysis on an Agilent 1100 Series HPLC system equipped with a variable wavelength detector (280 nm). Fractionation was performed on XBridge Shield C18 column (10 × 100 mm, 3.5 μm bead size, Waters). Peptides were separated by their hydrophobicity at a high pH at a flow rate of 0.1 mL/min using a gradient of mobile phase A (5 mM ammonium formate, pH 10) and a mobile phase B (100% ACN), from 3% to 35% over 60 mins. Fractions were collected every minute across the gradient length and concatenated into 24 fractions. Eluted peptides were dried in a SpeedVac centrifuge and reconstituted in MS loading buffer (2% ACN/0.1% FA) prior to MS analysis.

#### 2.5.3 Mass spectrometry and data analysis

Peptides were separated by reverse‐phase chromatography on a 1.6 μm C18 fused silica column (ID 75 μm, OD 360 μm × 25 cm length) packed into an emitter tip (IonOpticks, Australia), using a nano‐flow HPLC (M‐class, Waters). The HPLC was coupled with an Impact II UHR‐QqTOF mass spectrometer (Bruker, Bremen, Germany) using a CaptiveSpray source and nanoBooster at 0.20 Bar using acetonitrile. Peptides were loaded directly onto the column at a constant flow rate of 400 nL/min with buffer A (99.9% Milli‐Q water, 0.1% formic acid) and eluted with a 90 minutes linear gradient from 2% to 34% buffer B (99.9% ACN, 0.1% FA). Mass spectra were acquired in a data‐dependent manner including an automatic switch between MS and MS/MS scans using a 1.5‐second duty cycle and 4 Hz MS1 spectra rate followed by MS/MS scans at 8‐20 Hz dependent on precursor intensity for the remainder of the cycle. MS spectra were acquired between a mass range of 200‐2000 m/z. Peptide fragmentation was performed using collision‐induced dissociation (CID).

Raw files consisting of high‐resolution MS/MS spectra were processed with MaxQuant (version 1.5.8.3) for feature detection and protein identification using the Andromeda search engine.^32^ Extracted peak lists were searched against the UniProtKB/Swiss‐Prot Mus musculus or Homo sapiens databases (October 2016) and a separate reverse decoy database to empirically assess the false discovery rate (FDR) using strict trypsin specificity allowing up to two missed cleavages. The minimum required peptide length was set to seven amino acids. In the main search, precursor mass tolerance was 0.006 Da and fragment mass tolerance was 40 ppm. The search included variable modifications of oxidation (methionine), amino‐terminal acetylation, the addition of pyroglutamate (at N‐termini of glutamate and glutamine), and a fixed modification of carbamidomethyl (cysteine). The “match between runs” option in MaxQuant was used to transfer identifications made between runs on the basis of matching precursors with high mass accuracy.^33^ Protein abundance was calculated using the intensity‐based absolute quantification (iBAQ) metric.^34^ Peptide‐spectrum matches (PSMs) and protein identifications were filtered using a target‐decoy approach at an FDR of 1%. Protein identification was based on a minimum of one unique peptide. The mass spectrometry proteomics data have been deposited to the ProteomeXchange Consortium via the PRIDE partner repository^35^ with the dataset identifier PXD014333 and the following Username: reviewer23562@ebi.ac.uk and Password: A7eE5hnQ.

#### 2.5.4 Label‐free quantitative proteomics pipeline

Statistically relevant protein expression changes between the DTX and control mouse TIF samples were identified using the default workflow in the R package Proteus (version 0.2.10) where quantitation was performed at the peptide level, with some minor differences.^36^ Only unique and razor peptides were considered for quantification with intensity values present in at least two out of three replicates per group. Missing values were replaced by values drawn from a normal distribution of 1.8 standard deviations and a width of 0.3 for each sample (Perseus‐type). Peptides were assigned to their leading razor protein and peptide intensities were aggregated to protein intensities using the aggregateHifly function based on the high‐flyer method. Peptides were assigned to their leading razor protein and peptide intensities were aggregated to protein intensities using the aggregateHifly function based on the high‐flyer method.^37^ Protein intensities were normalized according to the normalize Quantiles function from the limma Bioconductor package.^38^ Differential protein expression was performed using the limmaDE function which uses the empirical Bayes moderated *t* tests using the limma package. Protein intensities were log2 transformed. Proteus corrects for multiple testing using the Benjamini‐Hochberg FDR procedure.

### 2.6 Analysis of mouse TIF protein localization

The gene symbol of each TIF protein was interrogated in an RNAseq dataset from normal mice and from those with adult germ cell ablation using the germ cell‐specific toxicant busulfan.^39^ Proteins were deemed to be predominantly expressed in germ cells when their mRNA levels were decreased in whole testes by >70% after busulfan treatment. The genes corresponding to TIF proteins were also interrogated in a microarray dataset of isolated seminiferous tubule cells (Sertoli cells, spermatogonia, pachytene spermatocytes, or round spermatids), whole testes, and 18 other normal mouse tissues.^40^ Proteins were deemed likely to be contributed to TIF by adluminal germ cells when: (a) the protein was significantly reduced (*P* < .05) in TIF by DTX; (b) the mRNA was reduced >70% in whole testes by germ cell ablation,^39^ (c) their mRNA was >5‐fold enriched in isolated adluminal germ cells (PSC and/or rST) compared to Sertoli cells^40^ and (d) their mRNA was either undetectable (<6.50 log2 expression) or low (<7.64 log2 expression) in isolated Sertoli cells. The relative expression of these proteins in adluminal germ cells and testis was also compared to a range of normal mouse tissues.^40^

### 2.7 Quantitative Western blotting

Western blotting was performed for the quantification of LDHC in human testicular interstitial fluid from men with normal spermatogenic function (OA) and those with infertility and a presumed phenotype of Sertoli cell‐only (SCO). TIF samples containing 50 µg of protein were mixed with 6:5 (v/v) NuPAGE LDS Sample Buffer (4X) and 3:1 (v/v) of NuPAGE Sample Reducing Agent (10X). Proteins were resolved on a 4%‐12% Tris‐Glycine gel, at 110V for 90 min, using the Invitrogen min‐gel system. After transfer onto an Immobilon‐P PVDF membrane (Merck KGaA, Germany), proteins were fixed by drying at 37°C for 20 min. LDHC was detected using a rabbit polyclonal antibody (Invitrogen, Cat Nr PA5‐30079) and the IRDye 800CW Goat anti‐Rabbit IgG Secondary Antibody (Cat Nr 326‐32211; LI‐COR, NE, USA) diluted 1:5000 and 1:10 000, respectively, in the Odyssey Blocking Buffer (PBS) (LI‐COR). The blot was imaged in an Odyssey Fc Imaging System and the intensity of the bands was measured after a 2‐min exposure time by Image Studio Quantification Software (LI‐COR) and expressed in arbitrary units. Statistical differences between groups were determined using the Mann‐Whitney test on GraphPad Prism 8.4.2. Blots were performed on the same samples three times.

## 3 RESULTS

### 3.1 Defining the contribution of the seminiferous epithelium to the mouse TIF proteome

**FIGURE 1:**
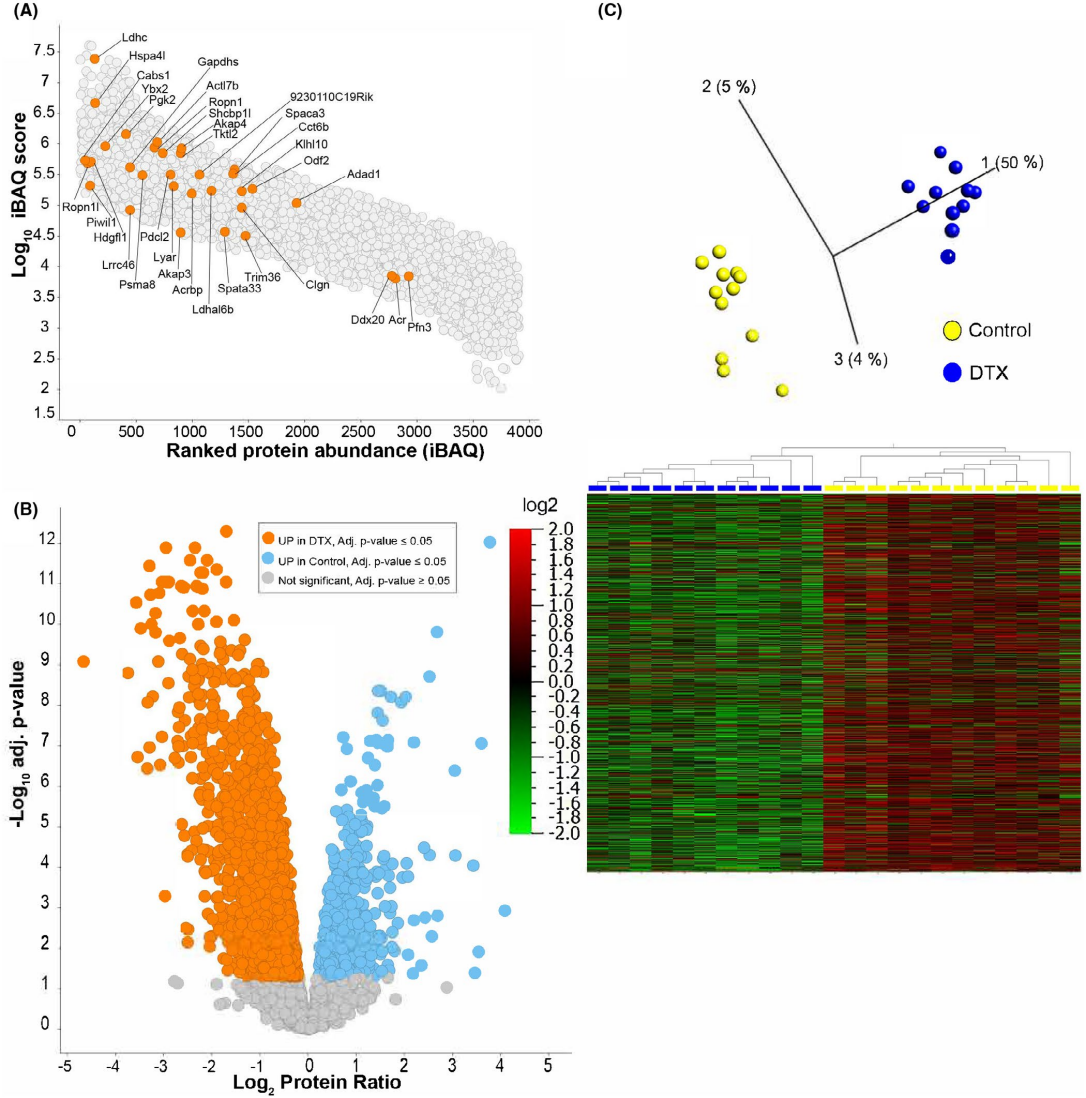
Impact of DTX‐treatment on the mouse TIF proteome. A, iBAQ analysis and dynamic range estimation of all quantified proteins in TIF from normal mice with intact spermatogenesis. iBAQ expression values for the proteins quantified are plotted with the log10 iBAQ intensity on the *y* axis and proteins are ranked by iBAQ intensity on the *x* axis. Highlighted proteins are those that are expressed specifically in adluminal germ cells in sperm in both mice and humans (see Table 1). B, Volcano plot illustrating the log_2_ protein ratios in mice with Sertoli cell ablation (DTX mice) relative to control mice. Significant differentially regulated proteins are colored in orange (UP in DTX treatment) or blue (UP in control mice) (adjusted *P* value ≤ .05). C, Principle Component Analysis (PCA, top) and heat map (bottom) of the n = 1812 proteins that were significantly different between control (yellow) and DTX (blue) TIF samples. The PCA plot reveals a clear separation between the control and DTX‐treated mice. Heat map data are log_2_ protein ratio in DTX mice relative to control

Testicular interstitial fluid was collected from mice using established methods,^24^, ^27^ and proteins and peptides were separated by reverse‐phase chromatography and identified by mass spectrometry (see Methods). A total of 3902 proteins were identified in TIF from normal (*control*) mice (Figure 1A, Supplemental Dataset 1A). To define the contribution of the seminiferous tubules to the mouse TIF proteome, we utilized a model of diphtheria toxin‐induced acute Sertoli cell ablation (*DTX mice*).^25^, ^26^ One week after DTX treatment, testes do not contain Sertoli cells, some advanced germ cells remain in the tubules but many are apoptotic, there is a marked decrease in spermatid marker gene expression (likely due to the induction of apoptosis), and the initial testicular inflammatory response to Sertoli cell ablation, as measured by inflammatory marker expression, has largely resolved (Supplemental Figures S1 and S2).^25^, ^26^

Using a label‐free quantitative proteomics pipeline, we quantified 3551 proteins in a comparative analysis of control vs DTX mouse TIF (Supplemental Dataset 1B). A total of 1812 TIF proteins were significantly different (*P* < .05) between control and DTX mice (Figure 1B). DTX caused a significant reduction in 1433 (41%) of TIF proteins (Figure 1B, Supplemental Dataset 1B and 2A) whereas n = 379 (11%) were up‐regulated (Figure 1B,C, Supplemental Dataset 2B). Two functional markers of peritubular myoid cells^26^ were present in TIF but not decreased in DTX mice (CNN1, MYH11, Supplemental Dataset 1B). This first in‐depth proteomic analysis of mouse TIF reveals that the seminiferous epithelium is a major determinant of the TIF proteome.

### 3.2 Germ cell proteins are present in mouse TIF

We next investigated TIF proteins that were decreased by DTX (Supplemental Dataset 2A). These included several well‐known sperm‐specific proteins such as ODF1, AKAP3, and AKAP4^41^, ^42^ that are thought to be restricted inside the blood‐testis barrier. If these proteins were present because of non‐specific leakage into TIF during fluid collection, we reasoned that most high‐abundance spermatid‐specific proteins should also be present. However, of the proteins whose mRNA is highly and specifically expressed in mouse spermatids but not detected in Sertoli cells (Supplemental Dataset 4A) or those with the highest expression in round spermatids (Supplemental Dataset 4B)^40^ only a subset was identified in TIF (Supplemental Dataset 4A,B) and only 39% of spermatid‐specific transcripts had protein products identified in TIF (Supplemental Dataset 4A). Furthermore, leakage should favor the release of germ cell proteins into TIF in DTX‐treated mice, where there is degeneration of the seminiferous epithelium, marked germ cell apoptosis, and disruption of tight junctions between Sertoli cells. However, we saw the opposite; sperm‐specific proteins ODF1, AKAP3, and AKAP4 were detected in high abundance in normal mouse TIF but were reduced ~90% after DTX‐induced seminiferous epithelial disruption (Supplemental Dataset 2A). These observations indicate some sperm‐specific proteins are deposited into TIF in the normal mouse testis.

To identify TIF proteins of germ cell origin, we investigated their testicular mRNA expression in an RNASeq dataset of germ cell ablation in mice.^39^ Proteins likely to originate from germ cells were identified using previously established criteria (a >70% reduction in mRNA expression in whole testis after the administration of the germ cell‐specific toxicant, busulfan).^39^ Of the 1433 proteins that were decreased in TIF after DTX treatment (Supplemental Dataset 2A), 498 were potential of germ cell origin (Supplemental Dataset 3A). These proteins showed a broad molecular weight range, from 5 to 541 kDa.

We further defined the cellular origin of these 498 proteins using mRNA data from purified mouse Sertoli and germ cells.^40^ Most were very highly expressed in germ cells, but also detectable in Sertoli cells. However, 141 TIF proteins were specifically expressed, or very highly enriched, in pachytene spermatocytes and round spermatids but were very low to undetectable in Sertoli cells and spermatogonia (Supplemental Dataset 3B). Thus, 141 proteins were highly enriched in germ cells that reside inside the blood‐testis barrier (ie, adluminal germ cells) and were significantly decreased in TIF by DTX ablation, suggesting that these are adluminal germ cell‐specific proteins contributed to TIF via Sertoli cells.

We next investigated which of these proteins were testis‐specific by assessing expression in a range of other adult male mouse tissues including brain, liver, and lung.^40^ Of the 141 spermatocyte and spermatid proteins, 95 had mRNA expression restricted to the testis (Supplemental Dataset 3B) and their abundance in TIF varied over a large range (Supplemental Dataset 3B). Therefore, at least 141 proteins are likely contributed to TIF solely by adluminal germ cells via Sertoli cells, and 95 of these proteins are only expressed in these cells and nowhere else in the adult mouse body.

Because sperm proteins often undergo translational delay during spermiogenesis^43^ it is likely that many of the TIF proteins with high mRNA expression in round spermatids (Supplemental Dataset 3B) are translated during the elongation phase of spermatids and are located in mature sperm. Consistent with this, germ cell proteins in TIF included well‐known components of spermatozoa, including components of the acrosome (ACRV1, SPESP1, SPACA3, and ZPBP^44^, ^45^, ^46^, ^47^), the fibrous sheath (AKAP3 and 4^41^), the outer dense fibers (ODF1 and 2^42^), the sperm flagellum (eg, SPA17, ROPN1, and ROPNL1^48^, ^49^), and chaperone proteins important for sperm function (eg, CALR3^50^).

We next investigated whether the appearance of a sperm protein in TIF was related to its location in spermatids (Supplemental Datasets 4B‐D). Proteins highly expressed in round spermatids and detected in TIF (Supplemental Dataset 4B) were significantly associated with sperm flagellar and cytoplasmic functions (Supplemental Dataset 4D), whereas those not detected in TIF were significantly enriched in nuclear/nucleosome functions and mitochondria (Supplemental Datasets 4B,C). Of note, the highly abundant spermatid nuclear proteins PRM1 and TNP1 were not detected in TIF, yet sperm flagellar proteins such as ODF1 and 2, AKAP3 and 4, were identified (Supplemental Dataset 4B). These data suggest that sperm proteins are differentially deposited into TIF based on their cellular location.

The sperm‐specific protein LDHC has recently been shown to promote peripheral immune tolerance in mice, via interacting with immune cells outside of the seminiferous tubules.^23^ In contrast, another sperm protein, zonadhesin (ZAN), was non‐tolerogenic and thus assumed to remain within the seminiferous tubules.^23^ In support, we found LDHC was the fifth most abundant protein in mouse TIF (Supplemental Dataset 1A) but ZAN was not detected. LDHC showed a highly significant reduction in TIF after DTX treatment (*P* = 5.06 × 10^−8^), confirming that it is contributed to TIF by the seminiferous tubules in mice.^23^

Thus, mouse TIF contains hundreds of germ cell proteins, many of which are predominantly or only expressed in developing sperm that reside “inside” the blood‐testis barrier. These results identify a wide range of adluminal germ cell‐specific proteins that are selectively deposited into mouse TIF by Sertoli cells.

### 3.3 Identification of germ cell proteins in human TIF

**FIGURE 2:**
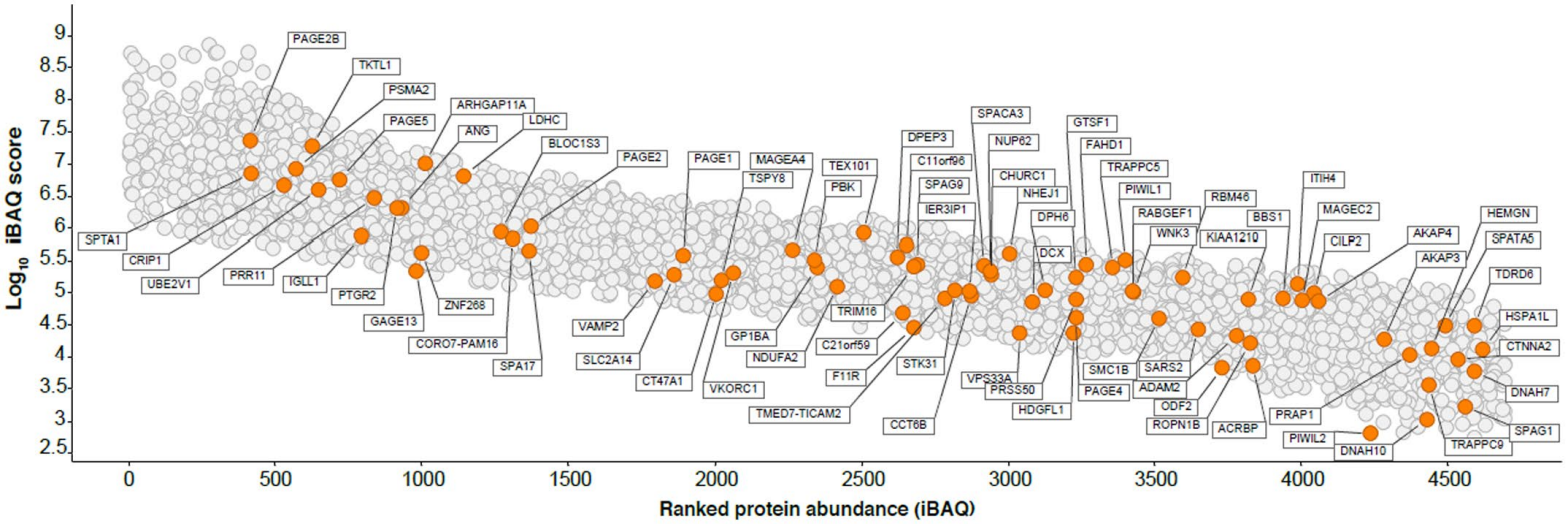
The human TIF proteome. iBAQ analysis and dynamic range estimation of n = 4720 proteins identified in human TIF. iBAQ expression values for the proteins quantified are plotted with the log10 iBAQ intensity on the *y* axis and proteins are ranked by iBAQ intensity on the *x* axis. Proteins that have been identified as cancer‐testis antigens (CTAs) are highlighted in orange (see Supplemental Dataset 8)

There is no data on the proteome of human TIF, and whether sperm‐specific proteins are also deposited into TIF by the seminiferous tubules in men is not known. Accordingly, we characterized the human TIF proteome from men with normal testis function. TIF was taken from three men undergoing surgical retrieval of sperm due to the obstruction of the distal reproductive tract (obstructive azoospermia, OA). These men had normal testis function based on a variety of clinical parameters (see Methods). It is important to note that the method of TIF collection in humans was somewhat different from that in mice (see Methods); however, both methods ensured that the seminiferous tubules remained intact. A total of 4720 proteins was identified in TIF from these men (Figure 2, Supplemental Dataset 5).

To identify proteins in human TIF that could arise specifically from germ cells, we utilized two different approaches. First, we looked for TIF proteins that are highly enriched in human spermatocytes and spermatids. We used an independent dataset of n = 1079 human testis‐enriched proteins (www.proteinatlas.org/humanproteome/testis 51), whose mRNA expression is enriched >5‐fold in human testis compared to all other tissues as assessed by RNASeq. This revealed 84 proteins in TIF whose expression is deemed to be enriched in human testis (Supplemental Dataset 6). The cellular localization of these human testis‐enriched TIF proteins was then further assessed using immunohistochemistry data in human testis sections (www.proteinatlas.org). We found 30 human TIF proteins that are highly enriched or specifically expressed in adluminal germ cells of the human testis (Supplemental Dataset 7A). This list included proteins involved in sperm motility (AKAP4, GAPDHS, LDHC, PGK2, and ROPN1L) and fertilization (ACR, ADAM2, CLGN). Twenty‐three of these proteins had orthologs that were present in mouse TIF and were decreased by DTX (Supplemental Dataset 7A).

In the second approach, we analyzed whether the 95 sperm‐specific proteins in mouse TIF (Supplemental Dataset 3B) have orthologs in human TIF and whether there is information supporting their enrichment or specificity in adluminal germ cells in men. Of these, 28 proteins had orthologs detectable in human TIF, and 21 have been shown to be specific to, or highly enriched in, human testis based on multiple human RNASeq‐based datasets (Supplemental Dataset 7B).

**FIGURE 3:**
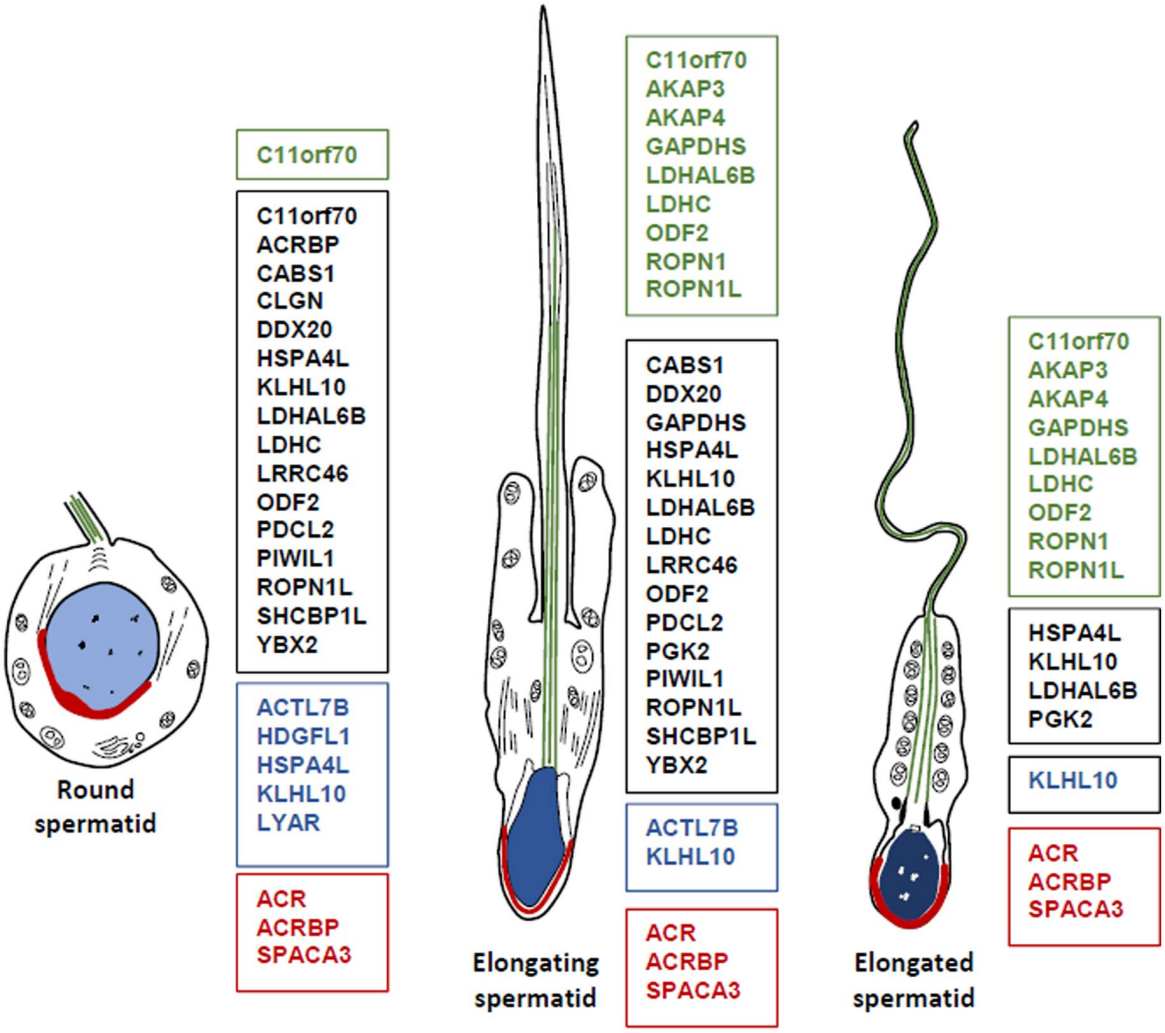
The localization of conserved spermatid‐specific TIF proteins in human spermatids. Diagram of a human round, elongating and elongated spermatid. The localization of sperm‐specific proteins found in both mouse and human TIF are indicated in the colored boxes and are grouped according to localization within spermatids based on human immunohistochemistry data (Human Protein Atlas); only TIF proteins with an immunohistochemistry reliability score of Enhanced or Supported are included. Different cytoskeletal protein localizations are indicated as follows: black = cytoplasm, blue = nucleus, green = flagellum, red = acrosome

Using the above data (Supplemental Dataset 7A,B), and relevant published datasets, we identified 33 TIF proteins that are likely to originate from adluminal germ cells in both mice and humans (Table 1, Figures 1A and 3, Supplemental Dataset 7C). All 33 proteins were significantly reduced in mouse TIF by DTX‐induced seminiferous epithelial disruption and had immunohistochemical and/or mRNA expression data to support their enrichment in adluminal germ cells in both species (Table 1, Figure 3, Supplemental Dataset 7C). These conserved TIF proteins include flagellar components (AKAP3 and 4), acrosomal proteins (ACR, ACRBP, SPACA3), and sperm‐specific enzymes (LDHAL6B, LDHC, GAPDHS) (Figure 3). These results demonstrate that the deposition of a range of sperm‐specific proteins into TIF is conserved in mice and humans.

**TABLE 1:** Adluminal germ cell proteins in TIF conserved between mice and humans^a^

Gene symbol (mouse, human)	Protein name (human)	Relative abundance (mouse)	Relative abundance (human)
9230110C19Rik, CFAP300/C11orf70	Cilia‐ and flagella‐associated protein 300, Uncharacterized protein C11orf70	+++	++
Acr, ACR	Acrosin	+++	++
Acrbp, ACRBP	Acrosin‐binding protein	++++	++
Actl7b, ACTL7B	Actin‐like protein 7B	++++	++
Adad1, ADAD1	Adenosine deaminase domain‐containing protein 1	+++	++
Akap3, AKAP3	A‐kinase anchor protein 3	++++	++
Akap4, AKAP4	A‐kinase anchor protein 4	++++	++
Cabs1, CABS1	Calcium‐binding protein, spermatid associated 1	++++	++
Cct6b, CCT6B	T‐complex protein 1 subunit zeta‐2	+++	++
Clgn, CLGN	Calmegin	+++	++
Ddx20, DDX20	DEAD‐box helicase 20	+++	++
Gapdhs, GAPDHS	Glyceraldehyde‐3‐phosphate dehydrogenase, spermatogenic	++++	++
Hdgfl1, HDGFL1	Hepatoma‐derived growth factor‐like 1	++++	++
Hspa4l, HSPA4L	Heat shock protein family A (Hsp70) member 4 like	++++	+++
Klhl10, KLHL10	Kelch‐like family member 10	+++	++
Ldhal6b, LDHAL6B	Lactate dehydrogenase A‐like 6B	++++	++
Ldhc, LDHC	Lactate dehydrogenase C	+++++	+++
Lrrc46, LRRC46	Leucine‐rich repeat‐containing protein 46	++++	++
Lyar, LYAR	Ly1 antibody reactive	++++	+++
Odf2, ODF2	Outer dense fiber of sperm tails 2	+++	++
Pdcl2, PDCL2	Phosducin‐like 2	++++	++
Pfn3, PFN3	Profilin‐3	+++	++
Pgk2, PGK2	Phosphoglycerate kinase 2	++++	++
Piwil1, PIWIL1	Piwi‐like protein 1	++++	++
Psma8, PSMA8	Proteasome subunit alpha type‐7‐like	++++	+++
Ropn1, ROPN1/ROPN1B	Rhophilin associated tail protein 1B	++++	++
Ropn1l, ROPN1L	Rhophilin associated tail protein 1 like	++++	++
Shcbp1l, SHCBP1L	SHC‐binding and spindle associated 1 like	++++	+++
Spaca3, SPACA3	Sperm acrosome associated 3	++++	++
Spata33, SPATA33	Spermatogenesis‐associated protein 33	+++	++
Tktl2, TKTL2	Transketolase‐like protein 2	++++	+++
Trim36, TRIM36	E3 ubiquitin‐protein ligase TRIM36	+++	++
Ybx2, YBX2	Y‐box‐binding protein 2	++++	+++

^a^ See Supplemental Dataset 7C for further details.

### 3.4 Sperm‐specific TIF proteins can be detected in human blood plasma

Because proteins in TIF can enter the circulation,^52^ we next investigated whether these advanced germ cell‐specific human TIF proteins could be detected in human plasma by performing in silico analysis of the Plasma Proteome Database.^53^, ^54^ We found that 22 human sperm‐specific TIF proteins are detected in human plasma; ACR, ACTL7B, ADAM2, AKAP3, AKAP4, CABS1, CCT6B, CLGN, DDX20, GAPDHS, HSPA4L, LDHAL6B, LDHC, ODF2, PFN3, PGK2, PRSS50, PSMA8, SHCBP1L, TRIM36, TTC21A, YBX2 (Supplemental Dataset 7A,C). These proteins are also testis and germ cell‐specific in mice (Supplemental Dataset 7C). These findings contradict the widely held assumption that sperm‐specific proteins do not have access to the circulation and suggest that sperm proteins released into TIF can enter the circulation.

### 3.5 Cancer‐testis antigens are present in mouse and human TIF

We noted several well‐known cancer‐testis antigens (CTAs) were detected in both mouse and human TIF. These are proteins whose normal expression is restricted to advanced germ cells/sperm but are aberrantly expressed in cancer.^7^, ^8^, ^10^, ^15^ We investigated human CTAs in TIF utilizing published datasets,^8^, ^55^, ^56^ including a recent study identifying germ cell‐specific CTAs in humans.^56^ Human TIF contained 82 proteins that are classified as CTAs (Figure 2, Supplemental Dataset 8). Of these, 43 are detected in human plasma according to the Plasma Proteome Database (Supplemental Dataset 8), challenging the assumption that germ cell‐specific CTAs do not appear in the circulation.

Furthermore, 41 of the 82 human TIF CTAs have orthologs detectable in mouse TIF (Supplemental Dataset 8). Of these, 31 showed significant decreases in mouse TIF after DTX treatment, indicating that they are contributed to TIF by the Sertoli cells in mice. These proteins included well known sperm‐specific CTAs that are being explored for their utility as cancer treatments and/or biomarkers, including AKAP3 and 4, ACRBP (also known as OY‐TES‐1), and SPACA3.^57^, ^58^, ^59^, ^60^ We also detected several human‐specific CTAs in TIF, including MAGEA4 and MAGEC2, as well as various PAGE CTAs (eg, PAGE1, PAGE2B, Supplemental Dataset 8).

### 3.6 A germ cell‐specific protein and CTA is significantly reduced in TIF from infertile men

In order to further verify the existence of a sperm‐specific protein and CTA in human TIF using a technique independent of mass spectrometry, we used Western blot analysis. We selected the TIF protein LDHC as it is expressed only in elongated spermatids and can promote peripheral tolerance in mice^23^ and is highly abundant in mouse TIF (Table 1). LDHC is also a CTA and has been suggested to be a promising target for immunotherapy.^61^ We also chose LDHC because it shows a higher relative abundance in both mouse and human TIF compared to other sperm‐specific proteins and thus is more likely to be detectable by the less sensitive technique of Western blotting.

**FIGURE 4:**
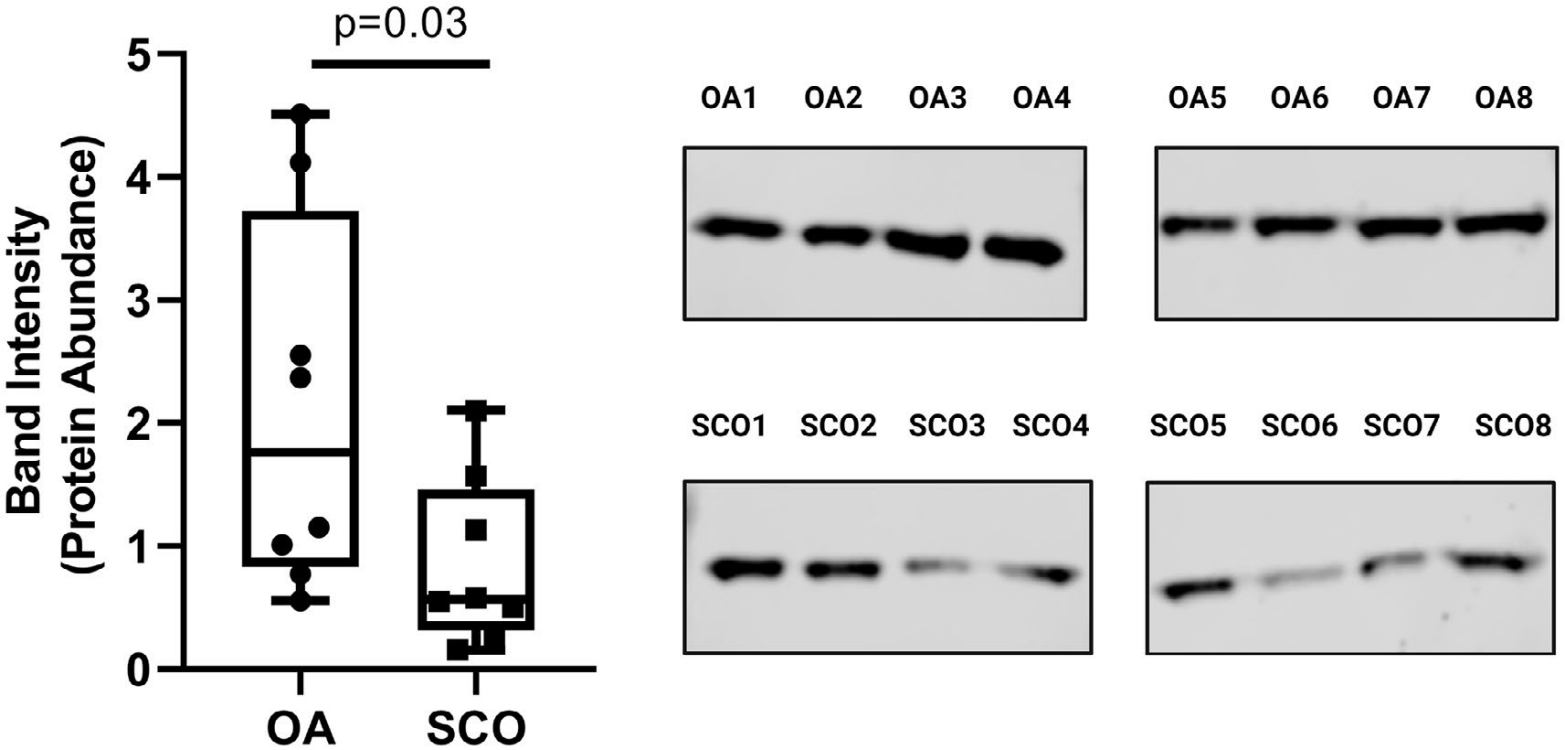
Western blot analysis of a sperm‐specific protein and CTA in TIF from men with normal vs abnormal spermatogenesis. Western blotting for LDHC was performed on 50 µg of TIF protein collected from n = 8 men with normal spermatogenesis based on testis biopsy (Obstructive Azoospermia, OA) and n = 8 men with a phenotype of presumed Sertoli cell‐only (SCO), based on a spermatogenic score of 0; these men were unable to have sperm retrieved during microsurgical‐assisted testicular sperm extraction (see Methods). Blots were scanned and statistical differences in band intensity between groups were calculated by the Mann‐Whitney test. The experiment was repeated three times and a statistically significant difference with a *P* value of .03 was observed between OA and SCO samples in all three experiments. A representative experiment is shown here with the box plot indicating median, 1st and 3rd quartiles, and data points from individual patients

Immunoreactive LDHC protein was detectable in TIF samples from eight men with normal spermatogenesis who underwent testis biopsy due to obstructive azoospermia (Figure 4); these samples are from a separate cohort of patients to those used in the mass spectrometry study.

Importantly, LDHC levels were significantly reduced in men with nonobstructive azoospermia (Figure 4, *P* = .03, n = 8/group). The phenotypes of these men were broadly classified as Sertoli cell‐only (SCO) based on testicular biopsy (Supplemental Dataset 10A,B), and when their testes were biopsied for the purpose of sperm retrieval, no sperm were retrieved. However, the variation in the levels of LDHC levels, ranging from very low to similar to those with obstructive azoospermia, suggests there could be varying levels of spermatid development in the seminiferous tubules of these individuals (Figure 4). These results are the first to demonstrate that the levels of a sperm‐specific protein and CTA in human TIF vary according to the status of spermatogenesis.

## 4 DISCUSSION

**FIGURE 5:**
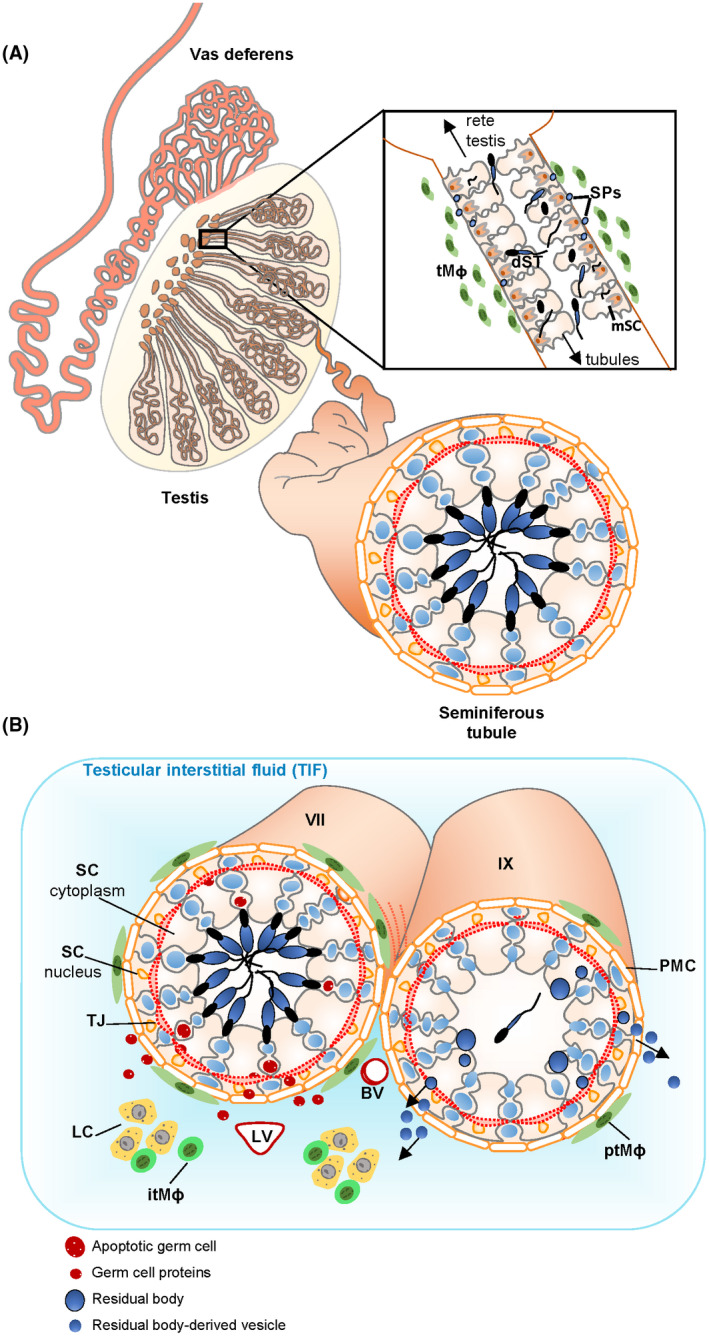
Model of sperm protein deposition into TIF. A, Diagram of the sperm‐producing seminiferous tubules that are coiled inside the testis, *Inset*: the tubuli recti are short segments of modified tubules at the end of the seminiferous tubules as they terminate at the rete testis. These tubules contain a narrow lumen and an epithelium of modified Sertoli cells (mSC). Fragments of degenerating sperm (dST) often appear in the epithelium and sperm proteins (SPs) are hypothesized to exit the tubules due to a weak epithelial barrier.^77^ MHCII+ testicular macrophages (tMɸ) are concentrated around this site in the normal testis. B, The seminiferous tubules are surrounded by basement membrane and peritubular myoid cells (PMC); inside the tubules, the seminiferous epithelium is comprised of the somatic Sertoli cells (SC) and the male germ cells (shown in blue) at various stages of development. Tight junctions (TJ) between Sertoli cells prevent the free passage of molecules into and out of the tubules. Thus, the inner‐tubule milieu is strictly determined by the seminiferous epithelium; this site is known as the adluminal compartment of the tubules. The testicular interstitial fluid (TIF) surrounds the tubules; the interstitium contains the steroidogenic Leydig cells (LC), blood vessels (BV), lymphatic vessels (LV), and spherical, MHCII‐ interstitial testicular macrophages (itMɸ). Elongated MHCII+ peritubular macrophages (ptMɸ) surround the seminiferous tubules, with the number of macrophages varying according to the stages of germ cell development.^65^ After mature sperm are released from the tubules, remnants of their cytoplasm known as residual bodies are phagocytosed and processed; some of the contents of these residual bodies are proposed to be released in residual body‐derived vesicles from stage IX tubules (IX) and to promote peripheral tolerance.^23^ We hypothesize that germ‐cell proteins could also be released from stage VII tubules (VII) where there is continual apoptosis of a small percentage of spermatocytes and round spermatids. MHCII+ peritubular macrophage numbers are highest around this stage^65^

This study identifies germ cell and sperm‐derived proteins that are present in testicular interstitial fluid, outside the seminiferous tubules, in mice and humans. An understanding of whether, and which, germ cell proteins are deposited outside of the blood‐testis barrier in humans is important, because the assumption that germ cell‐specific proteins are restricted within the blood‐testis barrier underpins the concept that certain CTAs are neoantigens and ideal targets for cancer immunotherapy or as biomarkers for cancer surveillance.^7^, ^8^, ^10^, ^56^, ^62^, ^63^ Reproductive biologists have long speculated not all sperm‐derived proteins are sequestered within the seminiferous tubules^6^, ^17^, ^18^, ^19^, ^20^, ^64^ and in mice, a sperm‐specific protein, LDHC, can promote peripheral immune tolerance,^23^ suggesting that it is not sequestered. We previously detected a small number of germ cell proteins in rat TIF using a lower resolution proteomic method^24^; however, because these proteins were also expressed elsewhere we could not establish whether they arose from the seminiferous tubules. The current study compared high‐resolution TIF proteomes from mice with and without a functional seminiferous epithelium, and compared fertile mouse and human TIF proteomes, to reveal that a conserved feature of the testis is the deposition of numerous germ cell and sperm‐derived proteins into TIF. Mass spectrometry resolved 3902 and 4720 proteins in mouse and human TIF, respectively. We annotated many of these proteins according to their localization in the testis and elsewhere. We identified at least 95 proteins in mouse TIF that are only expressed in germ cells residing inside the blood‐testis barrier, and our in vivo model suggests these proteins are contributed to TIF by Sertoli cells. In human TIF, we identified 33 proteins that are specifically expressed in germ cells inside the blood‐testis barrier and 85 that are classified as CTAs. Finally, we used Western blotting of human TIF as an independent, non‐mass spectrometry‐based, method to detect immunoreactive human LDHC, a sperm‐specific protein, and CTA being considered as an immunotherapy target.^61^ LDHC was detectable in TIF from men with intact spermatogenesis, but was significantly reduced in men with nonobstructive azoospermia due to spermatogenic disruption. LDHC in TIF was also significantly reduced in mice with seminiferous epithelial disruption. Thus, our study identifies germ cell and sperm‐specific proteins, including CTAs, that enter TIF in the healthy adult male, and suggests that their deposition is dependent on Sertoli cell and seminiferous tubule function. In TIF, these proteins could interact with antigen‐presenting immune cells^19^, ^23^, ^65^, ^66^ and/or enter the circulation (Figure 5).

That not all sperm antigens remain sequestered within the tubules has long been speculated^6^, ^17^, ^18^, ^19^, ^20^, ^64^ as has the concept that peripheral tolerance to sperm antigens may be involved in the immune protection of these cells.^5^, ^18^, ^20^, ^67^ Proof‐of‐principle for these concepts in mice was provided by a study demonstrating an endogenous sperm‐specific protein (LDHC) can promote peripheral tolerance and can be detected in the testicular interstitial space in mice after the injection of an anti‐LDHC antibody.^23^ In support, we measured LDHC in testicular interstitial fluid from both mice and men and showed that it is has a high relative abundance, particularly in mice (5th and 749th most abundant protein in mouse and human TIF, respectively) and verified the presence of LDHC in human TIF by Western blotting. Thus, our study confirms that the release of a wide range of germ cell‐derived proteins by the seminiferous tubules is conserved between mouse and human, and identifies those proteins that are likely to originate specifically from germ cells and sperm in both species.

Not all proteins expressed in spermatids were detectable in mouse TIF; 61% of genes highly and specifically expressed in round spermatids^40^ were not detected in TIF by mass spectrometry, nor was the protein ZAN that has been previously suggested to be sequestered within mouse seminiferous tubules as it could not promote a peripheral immune response.^23^ Our analyses suggested that the appearance of a spermatid protein in TIF relates to its localization within the cell; cytoplasmic and flagellar proteins were more likely to be detected than nuclear proteins. We propose that the deposition of germ cell proteins into TIF is a selective process, likely dependent on the cellular location as well as the mechanism of entry, as summarized by our proposed model in Figure 5.

That a hitherto unrecognized range of germ cell‐derived proteins are deposited into TIF in mice and humans offers a new paradigm for cell‐cell communication within, and outside of, the testis, and suggests that germ cell proteins could regulate testis homeostasis. These germ cell‐derived proteins in TIF are accessible to resident immune cells in the interstitium (Figure 5). Macrophages with features of antigen‐presenting cells surround the mouse seminiferous tubules at specific stages of germ cell development^65^, ^66^ as well as at the tubuli recti where the seminiferous tubules terminate^19^ (Figure 5); such macrophages could be involved in the presentation of sperm antigens to Tregs in the testes or in the draining lymph nodes.^23^ The testis and epididymis have acquired multiple mechanisms to subdue the immune response to sperm antigens during male reproductive tract compromise, including local immune‐suppression mechanisms.^3^, ^5^, ^64^ Our data suggest the range of sperm antigens that could promote peripheral tolerance is much wider than previously understood^23^ and that peripheral tolerance to a spectrum of sperm proteins could provide another mechanism to prevent the induction of autoimmune inflammatory disorders of the reproductive tract.

Germ cell proteins in TIF could also have as yet unrecognized functions in cellular communication or testicular regulation. Interstitial macrophages influence steroidogenesis and local immune suppression^17^, ^68^ and bone marrow‐derived macrophages appear in the testis when germ cells appear^66^ surrounding the tubules at specific stages of germ cell development.^65^ Peritubular macrophages regulate the spermatogonial stem cell niche and acquire MHCII+ as germ cells mature.^65^, ^66^ Thus, testicular macrophages important for normal testis function could respond to the presence of germ cell‐derived proteins in TIF. There is also some evidence to suggest that germ cell proteins in TIF could be related to Leydig cell steroidogenesis. Although the presence of germ cells in the tubules is thought to play a minor role in steroidogenesis based on cell ablation models,^69^ germ cell transplantation into mice congenitally lacking germ cells stimulates steroidogenesis^70^ and Leydig stem cells differentially colonize the testis depending on whether germ cells are present.^71^ Thus, germ cell proteins, or proteins dependent on the presence of germ cells, in TIF could theoretically impact Leydig function/stem cell turnover in a manner that could impact lifelong spermatogenic potential; this could be particularly important in long‐lived species such as humans. Indeed, infertile men with poor spermatogenesis have worse Leydig cell function than fertile men after 15 years of follow‐up.^72^

Our in‐depth characterization of the mouse TIF proteome allows us to propose a model whereby the seminiferous tubules use multiple mechanisms to deposit a wide range of germ cell proteins into TIF (see Figure 5). Our mouse model demonstrates that protein deposition requires Sertoli cells, as sperm proteins showed major reductions in TIF when Sertoli cells were ablated by 1 week of DTX treatment, despite degenerating germ cells and spermatids remaining in the tubules. One likely mechanism is via Sertoli cell‐mediated deposition of residual bodies. Residual bodies are remnants of elongated spermatid cytoplasm removed prior to spermatid release and phagocytosed by the Sertoli cell.^73^, ^74^ A previous study speculated that residual bodies could transport LDHC out of the tubules to promote immune tolerance,^23^ and electron microscopy observations suggest that residual body‐derived lipid‐like particles may cross the Sertoli cell basement membrane and appear in the interstitium.^74^ We note mouse TIF contains proteins detected in residual bodies, such as LDHC, DNAJB3, RNF17, and SMCP, but other spermatid proteins present in TIF are not detected in residual bodies, eg, ACRV1,^46^ and AKAP4,^75^ suggesting additional mechanisms of deposition.

A second site of deposition could be the tubuli recti (also known as the transition zone), where the seminiferous tubules empty into the rete testis (Figure 5).^19^, ^76^, ^77^, ^78^ These are densely packed with atypical Sertoli cells^76^, ^78^ and interaction between spermatozoa‐derived antigens and the immune system at this site has been hypothesized.^76^, ^77^, ^78^, ^79^ We noted Sertoli cells of the tubuli recti are positive for *Amh‐Cre* and appear to be ablated in our model, supporting the concept that this site could be involved in sperm protein deposition into TIF.

A third mechanism could be via Sertoli cell‐mediated release of germ cell proteins in extracellular vesicles at the mid‐spermatogenic stages (stages VII‐VIII in mice and stages IV‐V in humans; these stages occur before residual body release), where there is significant stage‐specific apoptosis of pachytene spermatocytes and round spermatids^80^ (Figure 5). MHCII+ macrophages preferentially accumulate around these tubules,^65^ and could access antigens deposited by Sertoli cells. The mouse and human testis‐specific proteins in TIF, HDGFL1, and LYAR, are detected in round spermatids during these stages but are absent from mature sperm (Figure 3), suggesting that they are not deposited via residual bodies or the tubuli recti. Of interest, we observed that very few of the germ cell‐derived proteins in TIF had secreted annotations, but several mice and human TIF germ cell and sperm‐specific proteins have been detected in exosomes (Supplemental Dataset 7B). Based on these findings, we hypothesize that Sertoli cells deposit germ cell‐derived proteins into TIF via extracellular vesicles.

The detection of numerous germ cell‐derived proteins in mouse and human TIF has immediate relevance to the development of immunotherapies targeting CTAs in cancer treatment. The term CTA is used to describe proteins specifically expressed in male germ cells but activated in cancer.^8^, ^9^, ^10^, ^63^ Male germ cell‐specific genes are expressed in such a vast number of cancer types they have been suggested to be a hallmark of cancer^56^ and are emerging therapeutic targets, particularly for immunotherapy.^7^, ^62^ CTA‐based immunotherapy is often based on the assumption that the germ cell‐restricted expression means the protein is a neoantigen and a strong immune response could be harnessed to target CTA‐positive cancer.^7^, ^61^, ^62^ However, we show mouse and human TIF contains many germ cell proteins that could interact with antigen‐presenting macrophages outside the tubules^65^, ^66^ (Figure 5) and potentially promote Treg‐dependent peripheral tolerance as has been described in mice.^23^ Whether a germ cell protein in human TIF is a neoantigen will relate to its abundance, as has been suggested in mice,^23^ and to its innate immunogenicity. The abundance of a particular sperm protein in TIF will likely be related to the sperm‐producing capacity of an individual's seminiferous tubules. We confirmed that levels of a sperm‐specific protein in TIF, LDHC, was significantly lower in men with compromised spermatogenesis, providing the first evidence that the levels of a CTA in TIF could depend on the individual's fertility status. LDHC can promote peripheral tolerance in mice,^23^ and thus our study suggests that its ability to promote tolerance in humans would show sex‐ and fertility‐dependent differences. This is particularly important because LDHC is currently being considered as a target for cancer immunotherapy.^61^ Because there is wide variation in sperm counts between men, and men with congenital forms of infertility may never produce any sperm, it follows that there could be wide variation in tolerance to particular antigens across the entire male population, ie, a neoantigen in one man would not necessarily be a neoantigen in another. Thus, depending on the CTA being targeted, there could be wide variation in responses, both between individuals and between the sexes. Our data suggesting that some CTAs will not be equally immunogenic in all patients, due to some having prior exposure to the immune system, is critical to the design of future clinical trials aimed at targeting CTAs for cancer vaccine development. Our data also suggest that CTAs could show sex‐specific responses, another crucial consideration in the design of immunotherapies for different cancers. There are currently more than 39 cancer trials using CTAs registered in the Clinical Trials database (http://www.clinicaltrials.gov). While initial data suggest that CTAs are useful for stratifying patient outcomes for various tumor types, there are still issues with the induction of a sufficient immune response.^15^ Our findings strongly suggest that all CTAs should not be presumed to be neoantigens and that the measurement of a particular CTA in serum, levels of antibodies, and a comprehensive analysis of tissue expression,^56^ in a variety of men and women is necessary prior to determining its potential clinical utility.

CTAs are also being pursued as cancer biomarkers, as the protein (and antibodies to the protein) are assumed to be absent from circulation in healthy individuals.^15^, ^16^ Yet TIF proteins can readily enter the circulation via the peritubular lymphatics^52^ and we identified sperm‐specific proteins and CTAs in human TIF that have been detected in blood plasma by mass spectrometry.^54^ We suggest that some sperm‐specific CTAs can, and do, appear in the circulation, and could vary with spermatogenic capacity. Thus, the utility of a CTA as a biomarker needs to be assessed in a wide cross‐section of both men and women.

These findings provide new opportunities to monitor spermatogenesis by a non‐invasive blood test. A test to determine the spermatogenic capacity of the human testis would greatly advance the diagnosis and treatment of infertile men.^81^ Approximately 9% of infertile men do not produce enough sperm in the testis for a fertile ejaculate^82^ and, for most, surgical retrieval of sperm from their testes is their only prospect to achieve parenthood.^83^ The lack of markers to predict successful retrieval is a major issue in the management of these patients and success rates of retrieval vary according to the underlying cause^83^ with up to half of all patients having unsuccessful surgery.^29^ Our discovery that multiple germ cell‐specific markers are present in human TIF is a major step toward developing diagnostics to non‐invasively monitor spermatogenesis.

In conclusion, we reveal a conserved feature of the mouse and human testis is the deposition of a range of germ cell and sperm‐specific proteins into TIF. One function of these proteins could be to promote peripheral tolerance,^23^ which requires the constant priming of Tregs by antigens^18^ and which could be fulfilled by multiple mechanisms of antigen deposition into TIF as we have proposed (Figure 5). However, because the appearance of a multitude of sperm proteins in TIF has been hitherto unrecognized, there could be other roles for these proteins in male physiology. Our findings provide a sound rationale for the development of tests to non‐invasively monitor spermatogenic function. Our results also have major implications for CTA‐based cancer immunotherapy and biomarker development, including the potential for gender‐specific and male fertility‐related responses to particular CTA‐based treatments and diagnostic approaches.

## CONFLICT OF INTEREST

The authors have declared that no conflict of interest exists.

## AUTHOR CONTRIBUTIONS

L. O'Donnell, D. Rebourcet, L.B. Smith, and P.G. Stanton conceived and designed the study. L. O'Donnell wrote the manuscript, D. Rebourcet, L.F. Dagley, L.B. Smith, and P.G. Stanton contributed to writing and editing the manuscript. D. Rebourcet and L.B. Smith provided all mouse samples and data on the mouse model. R. Sgaier and D. Fietz provided data. L.F. Dagley, G. Infusini, and A.I. Webb provided proteomics expertise including the design, collection, and analysis of mass spectrometry data. L.F. Dagley performed the mass spectrometry, provided data, and wrote the Materials and Methods sections for the proteomics. L. O'Donnell analyzed the processed proteomic datasets. D. Rebourcet, J.M.D. Legrand, and R.M. Hobbs provided guidance and interpretation on mouse data. A. Pilatz, T. Diemer, and W. Weidner provided guidance, clinical data, and human samples. R.I. McLachlan provided guidance on clinical data interpretation. F. Chalmel provided mouse microarray data and advice and P.J. O'Shaughnessy provided mouse RNAseq data and advice. F. Chalmel, P.J. O'Shaughnessy, A. Pilatz, and T. Diemer edited the manuscript. L. O'Donnell, D. Rebourcet, and L.F. Dagley share the first authorship: D. Rebourcet and L.F. Dagley provided key data and analysis for different aspects of the study and participated in writing the manuscript, and L. O'Donnell was responsible for combining and analyzing all datasets and for writing the paper. The manuscript was approved by all authors.

## Supporting information

FIg S1‐S2

Supplementary Material

Supplementary Material

Supplementary Material

Supplementary Material

Supplementary Material

Supplementary Material

Supplementary Material

Supplementary Material

Supplementary Material

Supplementary Material
