# Supplementary material for: Sperm proteins and cancer‐testis antigens are released by the seminiferous tubules in mice and men
Source: FASEB J. 2021 Feb 10;35(3):e21397. doi: 10.1096/fj.202002484R (PMC7898903; doi:10.1096/fj.202002484R)

Supplemental Data Legends:

Supplemental Figure 1. Effects of 1 week of DTX-induced Sertoli cell ablation on the testis. A. Histology of the testis in control (vehicle) mice and after 1 week of DTX-induced Sertoli cell ablation (Dtx) showing that DTX treatment results in major degeneration of the seminiferous epithelium. Scale bar=100um. B. SOX9 (Sertoli cell-specific protein) immunostaining (green) in control (vehicle) vs DTX mice at 1 week (Dtx) demonstrates Sertoli cell ablation Scale bar=100um. C. Cleaved Caspase 3 staining of control (vehicle) vs DTX-treated mice for 1 week (Dtx) demonstrates massive germ cell apoptosis Scale bar=50um. D. Germ cell markers were analysed by qPCR; *Pouf5a1* (spermatogonia), Spo11 (spermatocytes) and *Tp1* (spermatids) were all significantly decreased following Sertoli cell ablation (Mann Whitney test n=6 vehicle and n=10 DTX, ***P<0.001, mean ± SEM).

Supplemental Figure 2. Effects of DTX-induced Sertoli cell ablation on inflammatory markers and testicular macrophages. A. mRNA expression of the Sertoli cell marker *Sox9* and the inflammatory cytokines *Il6* and *Tnfa* at 0 (n=6), 1 (n=2), 2 (n=2), 3 (n=2) and 7 (n=8) days of DTX treatment. B. mRNA expression of inflammatory markers after 7 days of DTX (Students t test n=5 vehicle and n=8 DTX, **P<0.01, ***P<0.001, mean ± SEM), logarithmic normalisation applied when required. C. Immunostaining of CD163-labelled macrophages after 0, 1, 2, 3 and 7 days of DTX treatment.

Supplemental Dataset legends:

Supplemental Dataset 1. The mouse TIF proteome. A. N=3902 proteins identified in TIF from control mice (n=12). B. N=3551 proteins quantified in TIF from control (n=12) and DTX mice (n=11).

Supplemental Dataset 2. Mouse TIF proteins significantly altered by DTX-induced Sertoli cell ablation. A. N=1433 proteins were significantly down-regulated (p<0.05) in TIF from DTX-treated mice vs controls. B. N=379 proteins were significantly up-regulated (p<0.05) in TIF from DTX-treated mice vs controls.

Supplemental Dataset 3. Mouse TIF proteins likely to arise from germ cells within the seminiferous tubules. A. N=498 proteins in mouse TIF that potentially arise from germ cells. B. N=141 mouse proteins that are deemed likely to be contributed to TIF by the adluminal germ cells of the mouse seminiferous epithelium.

Supplemental Dataset 4. Genes that are highly and specifically expressed in mouse spermatids and whether or not the protein were detected in TIF. A. Genes that are highly and specifically expressed in isolated mouse round spermatids and whether their protein was detected in TIF. B. Genes with highest mRNA expression in isolated mouse round spermatids and whether they were detected in TIF. C. DAVID functional clustering analysis of all probesets highly expressed in round spermatids but not detected in mouse TIF. D. DAVID functional clustering analysis of all probesets highly expressed in round spermatids and detected in mouse TIF.

Supplemental Dataset 5. The human TIF proteome. N=4720 proteins identified in human TIF.

Supplemental Dataset 6. Human TIF proteins that are enriched in the testis. Proteins in human TIF that are likely to be enriched in the human testis according to genomic and proteomic studies (Djureinovic et al, 2014, Mol Hum Reprod, 20(6):476 and www.proteinatlas.org).

Supplemental Dataset 7. Proteins in mouse and human TIF that likely arise from adluminal germ cells. A. Testis-enriched proteins in human TIF (from Supplemental Dataset 6) that are likely to be specifically expressed, or highly enriched, in the adluminal germ cells of the testis that are "inside" the blood-testis barrier, according to immunohistochemical data from Human Protein Atlas. B. Proteins in mouse TIF that are likely to be specifically expressed, or highly enriched, in the adluminal germ cells of the testis and whether or not they appear in human TIF. C. Proteins that are likely contributed to TIF by adluminal germ cells in both mice and humans. Data on cell specificity, tissue enrichment and immunolocalisation in Supplemental Datasets 7A and B and various databases was considered.

Supplemental Dataset 8. Cancer testis antigens (CTAs) identified in human TIF

Supplemental Dataset 9. Clinical data on n=3 Obstructive Azoospermia (OA) patients from which TIF was collected for proteomics analysis

Supplemental Dataset 10. Clinical data and testis biopsy results on Obstructive Azoospermia (OA) and presumed Sertoli cell only (SCO) patients from whom TIF was collected for Western blotting. A. Clinical data on OA and SCO patients. Values are median (range) for n=8 men per group and differences between groups was determined by

Mann Whitney test. B. Spermatogenesis score on individual testis biopsies taken from OA and SCO men.

Supplemental Figure 1

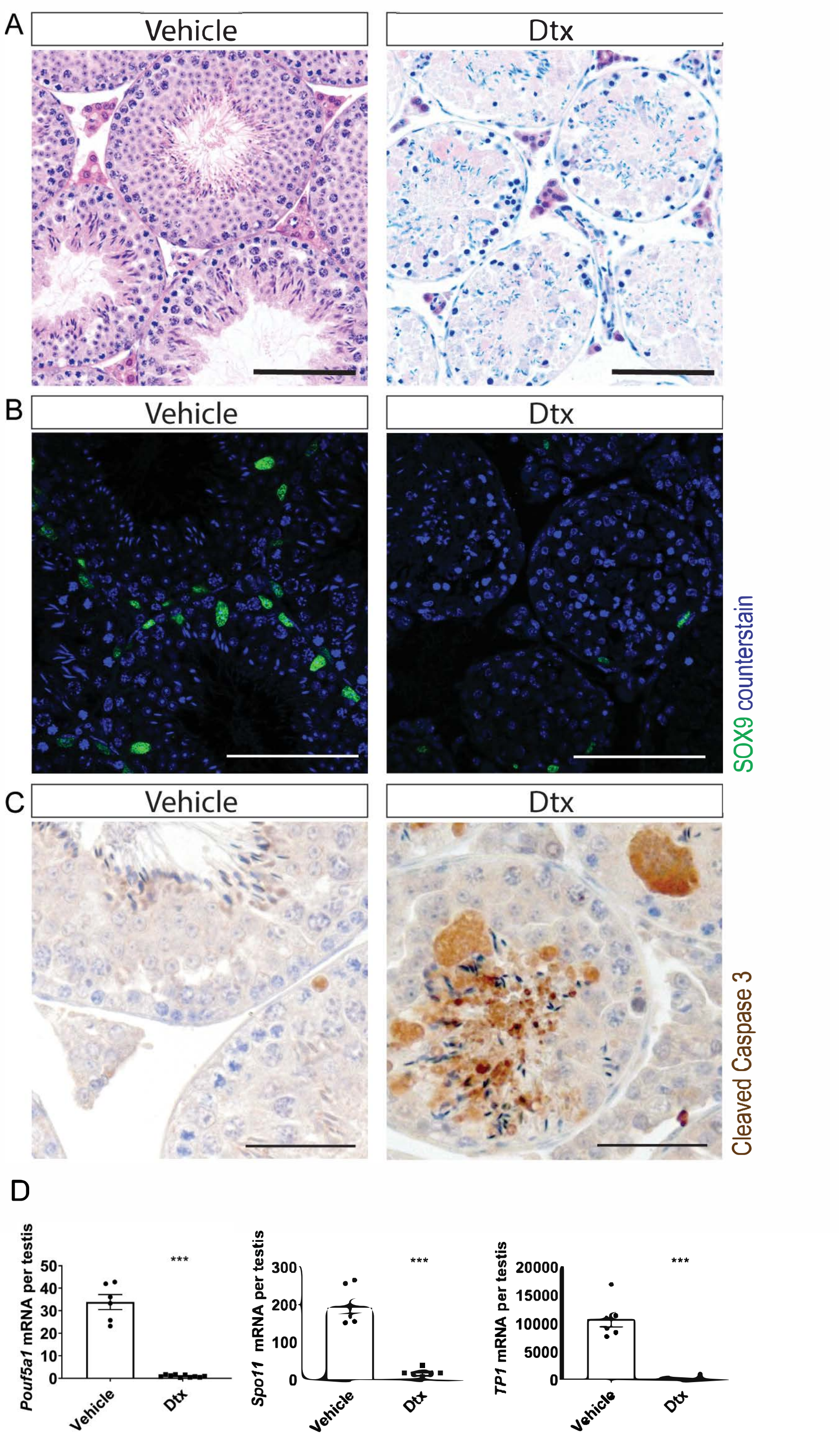

Supplemental Figure 2

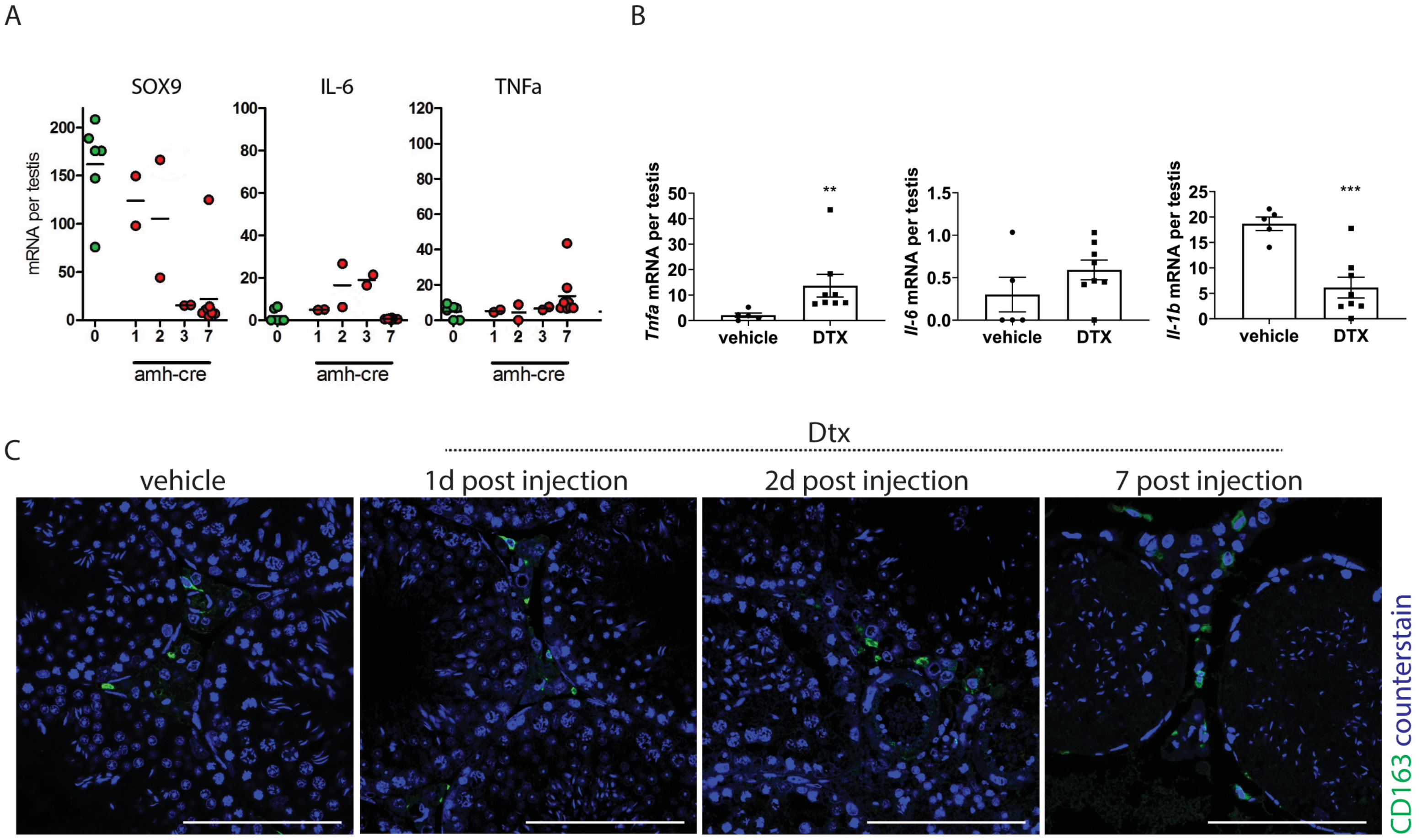

Supplement: Supplementary file 1 — FIg S1‐S2 [file FSB2-35-e21397-s008.pdf]
